# Supplementary material for: Functional analysis of N-acetylglucosaminyltransferase-I knockdown in 2D and 3D neuroblastoma cell cultures
Source: PLoS One. 2021 Nov 8;16(11):e0259743. doi: 10.1371/journal.pone.0259743 (PMC8575246; doi:10.1371/journal.pone.0259743)
Supplement: S1 Raw images — (PDF) [file pone.0259743.s001.pdf]

S1. Gelatinase of 3D samples

Figure 7C

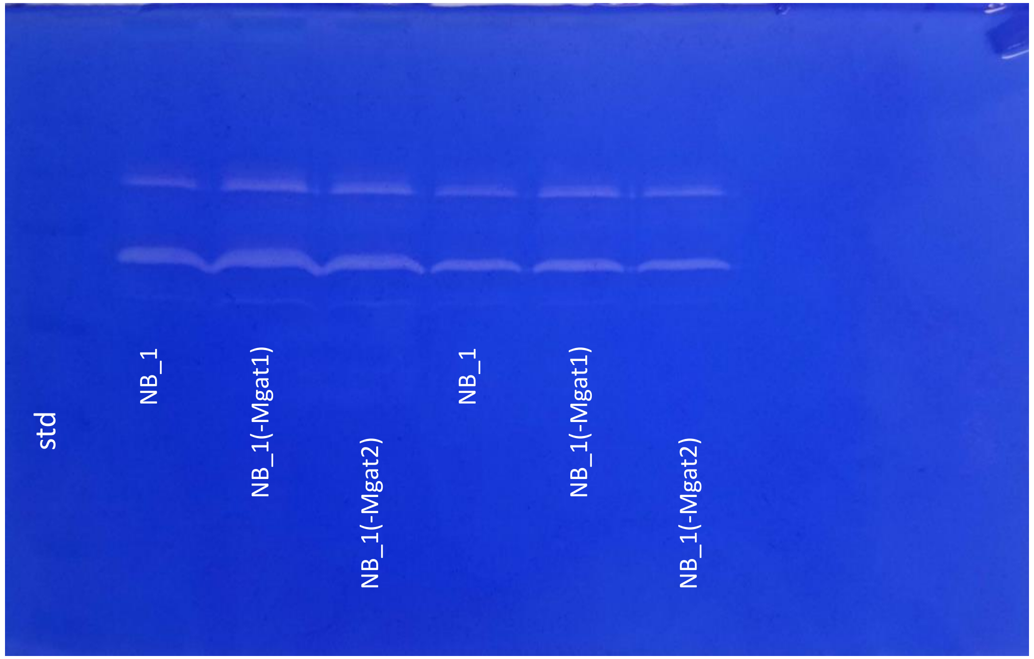

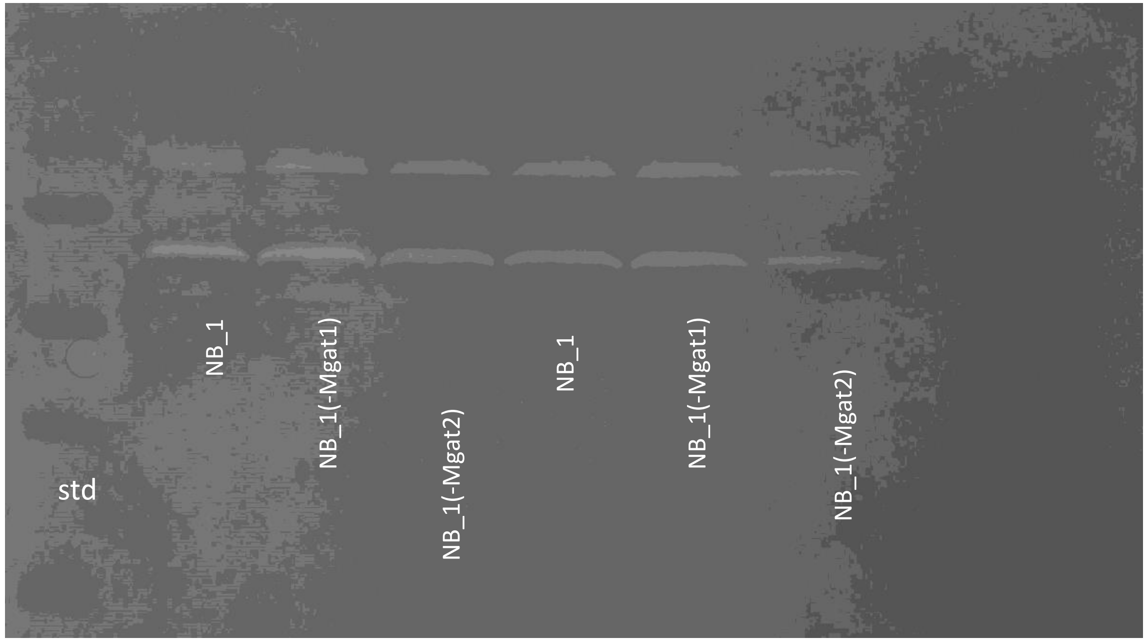

S2. Gelatinase of 2D samples

Figure 7A

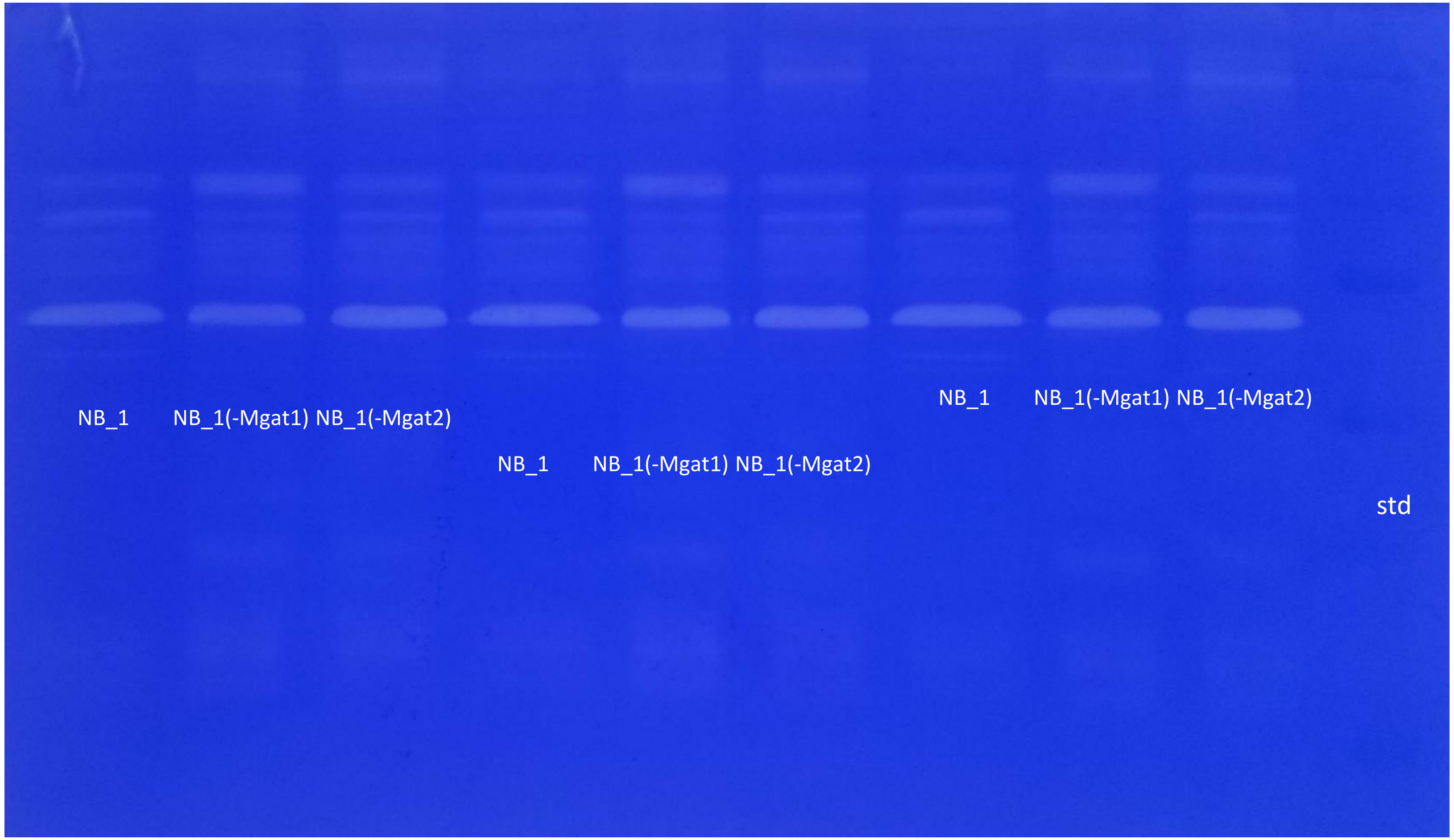

S3. HK2 detected in 2D and 3D samples

Figure 9A

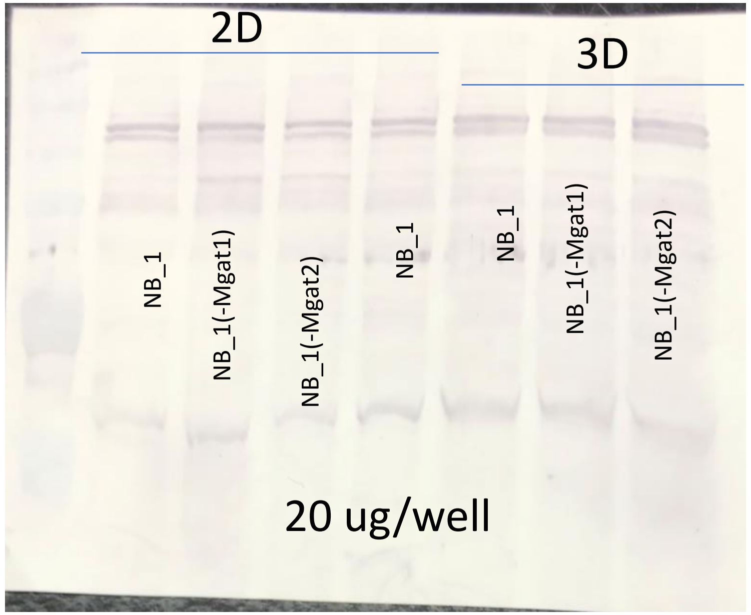

HK2

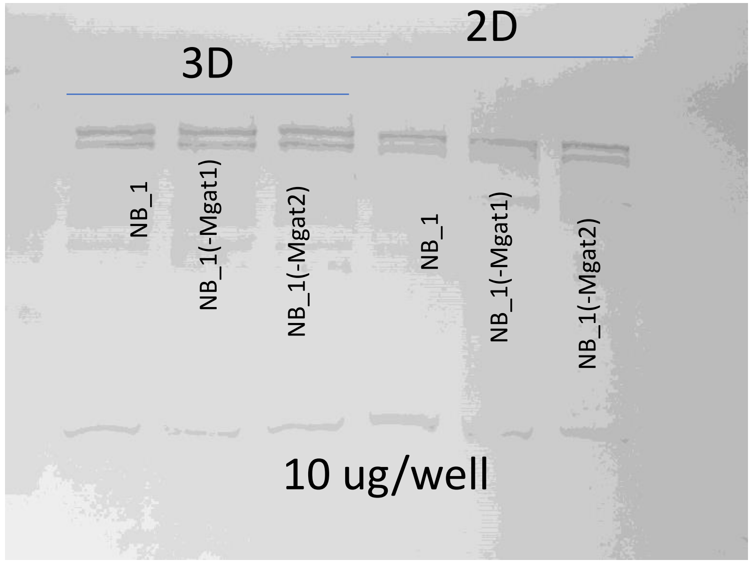

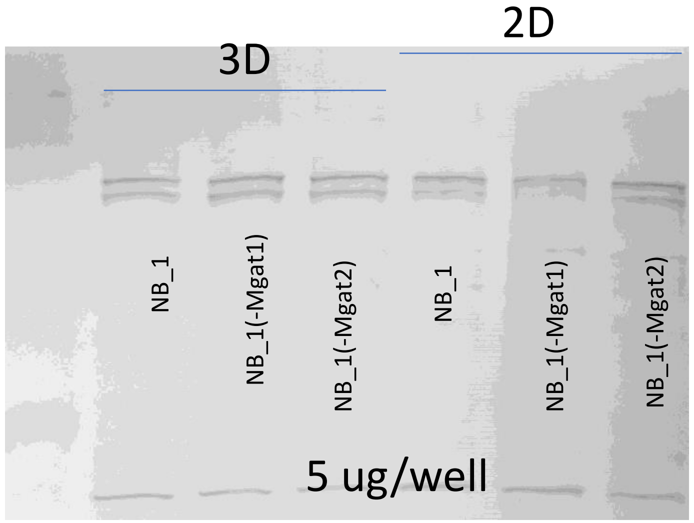

S4. PALLADIN detected in 2D and 3D samples

Figure 9A

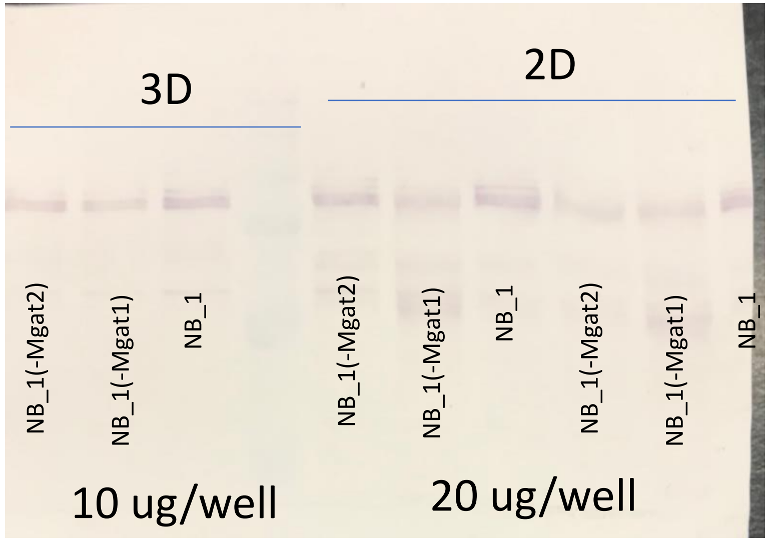

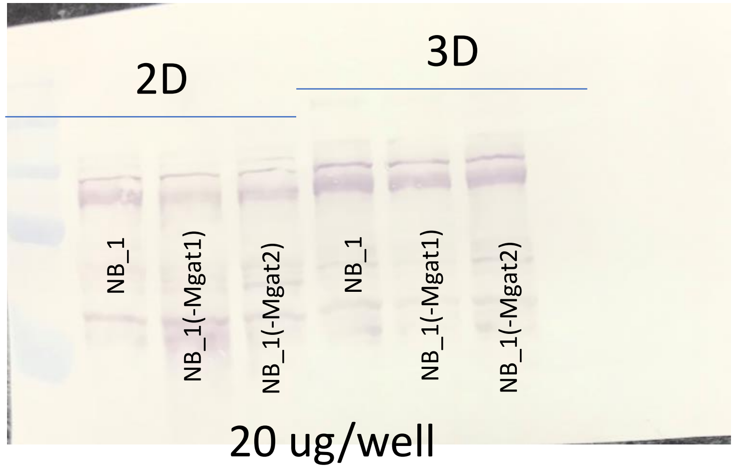

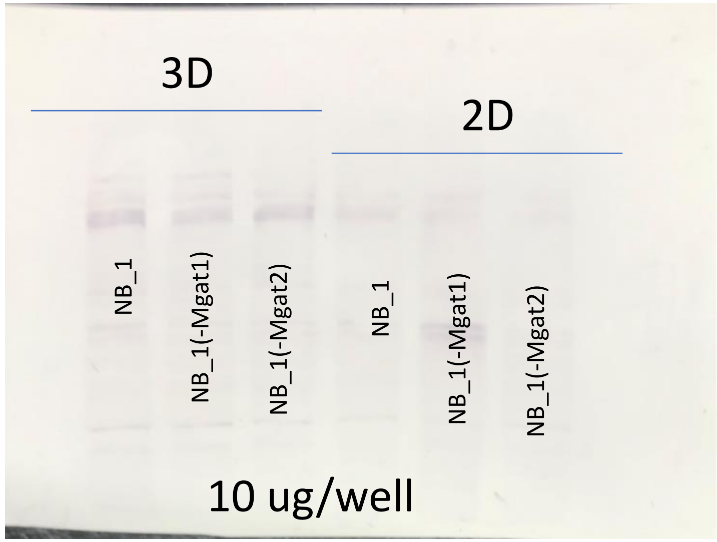

S5. Vimentin detected in 2D and 3D samples

Vimentin

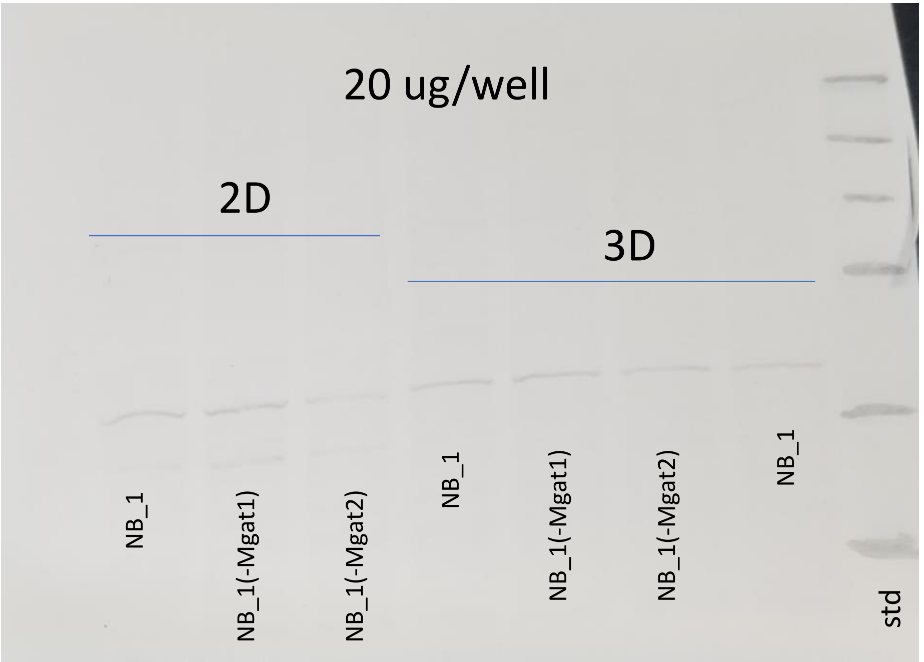

Figure 9A

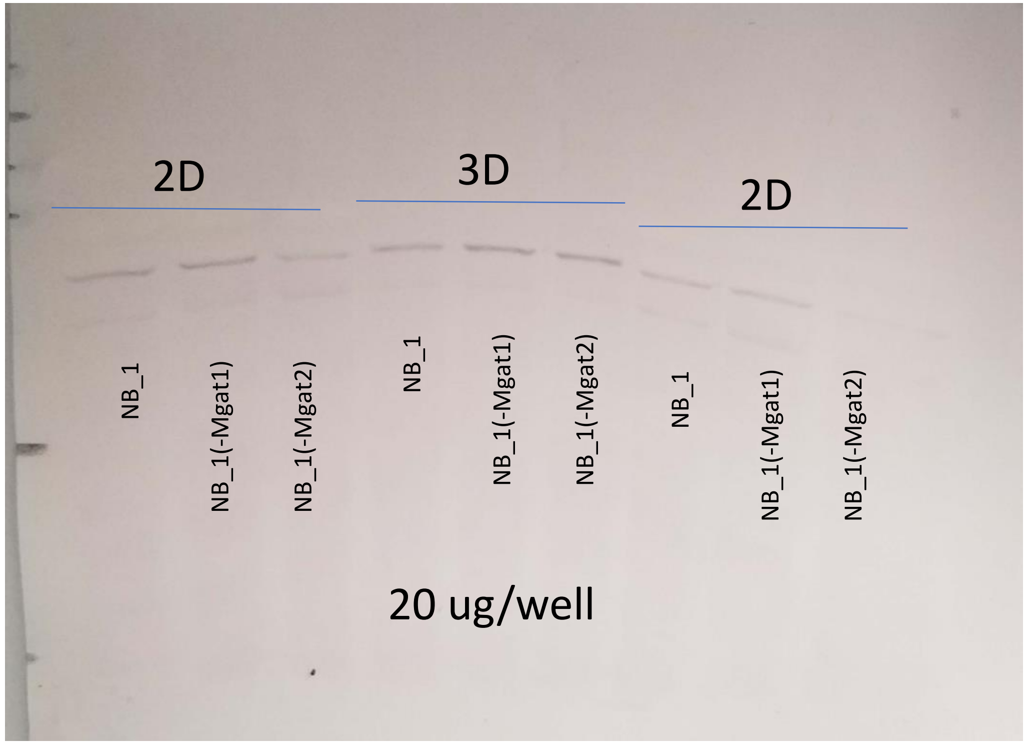

S6. EGFR detected in 2D and 3D samples

Figure 9A

2D 3D

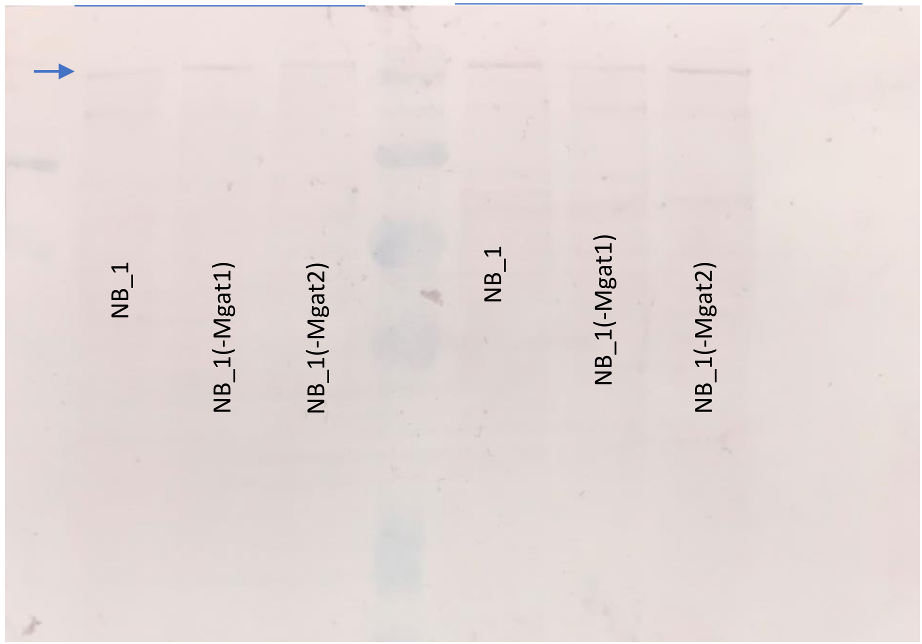

2D

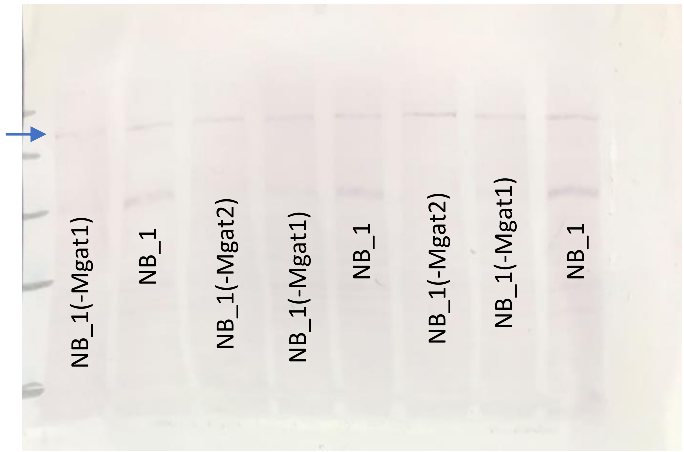

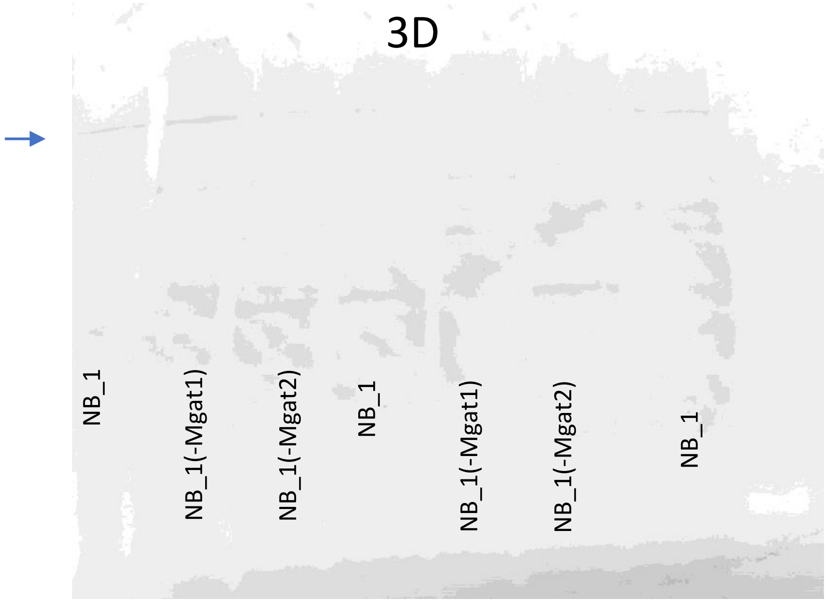

20 ug/well

S7. Coomassie Blue Stained Gels

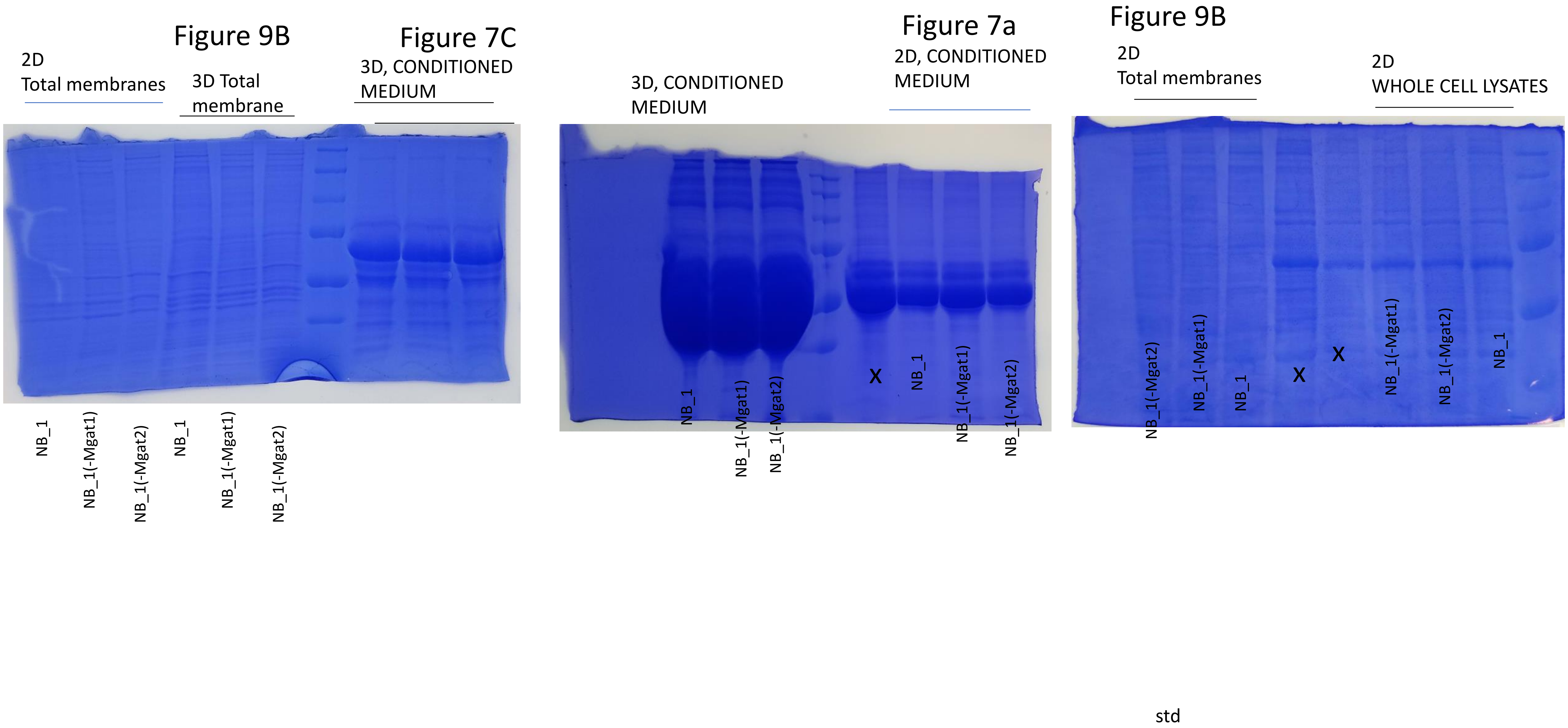

S7. Coomassie Blue Stained Gels, continued

Figure 9B

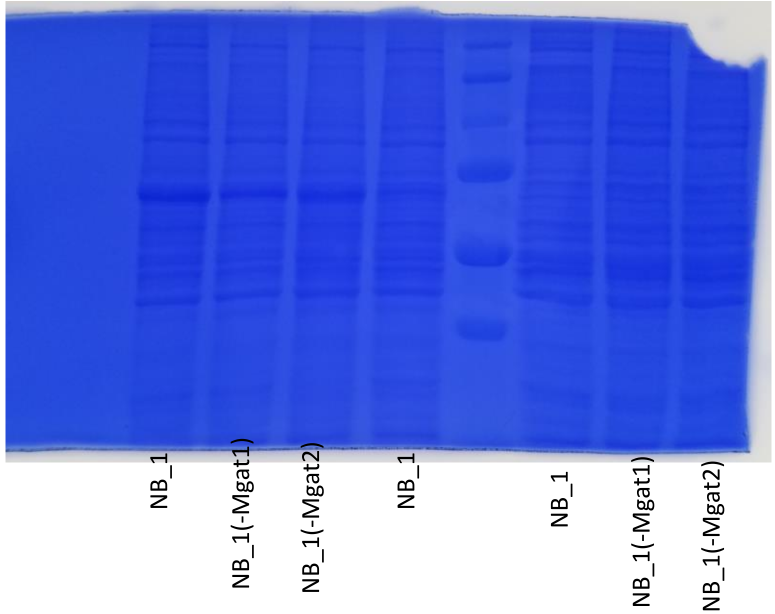

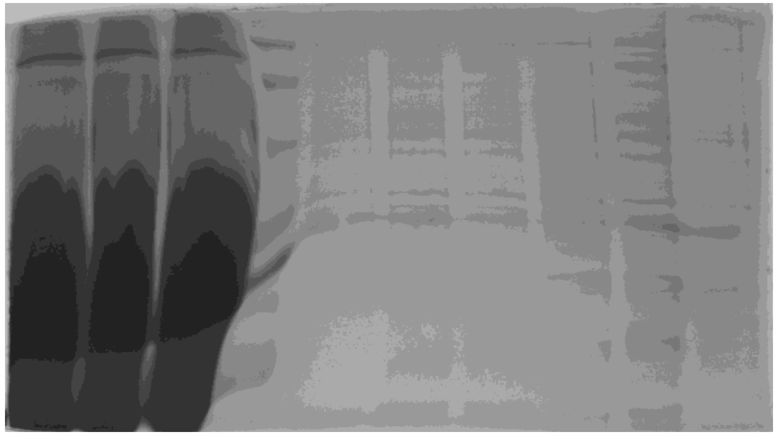

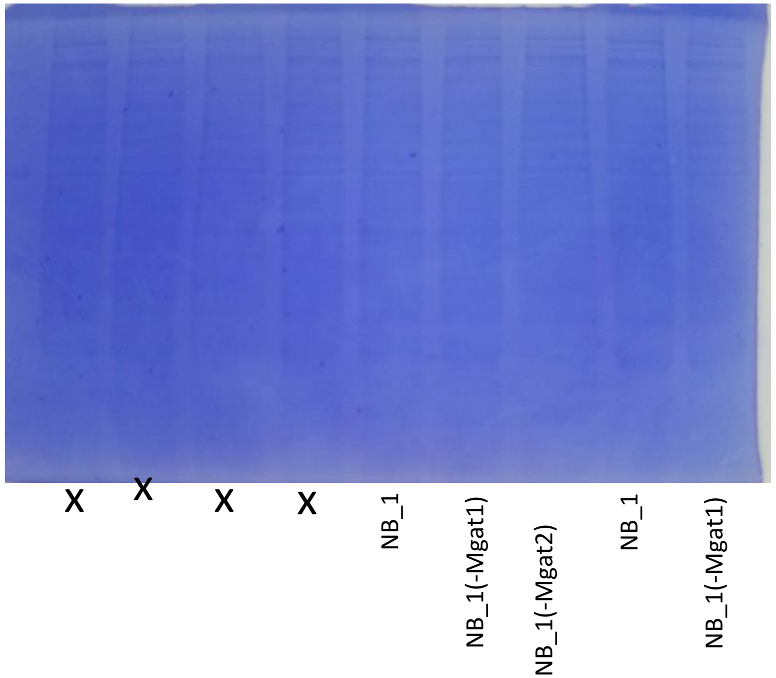

Images were captured using iphone or android cell phones
