# Supplementary material for: Functional analysis of N-acetylglucosaminyltransferase-I knockdown in 2D and 3D neuroblastoma cell cultures
Source: PLoS One. 2021 Nov 8;16(11):e0259743. doi: 10.1371/journal.pone.0259743 (PMC8575246; doi:10.1371/journal.pone.0259743)
Supplement: Attachment — Submitted filename: Point by point response to reveiwers.pdf [file pone.0259743.s003.pdf]

Point-by-point comments to reviewer

5. Review Comments to the Author

We wish to thank the reviewer for a thorough review as the comments have improved the manuscript.

Authors' answers to the questions above.

The answers to questions made by reviewer were positive. However, we have addressed availability of group data supporting the mean. Groups data has been uploaded as supplementary group data excel file. Also, we have attached a file: S1_Raw_images which has unmodified images of western blot, lectin blots, gelatinase gels and coomassie blue stained SDS gels.

Reviewer #1: In the manuscript by Hall et al., together with their previous paper (ref. 18-20) the authors generated Mgat1 (encodes GnT-I) knock out neuroblastoma cells by CRIPR/Cas9 and obtained mutant cells with aberrant N-glycosylation. The mutant cells showed altered cell morphology and growth properties compared to wild-type parental cells. The authors also showed that the expression of several proteins including MMP, receptor-tyrosine kinase and cytoskeletal proteins were significantly changed in mutant cells.
The experiment seems to be well designed and conducted, and the results ae convincing to support their conclusion. The manuscript is well constructed and written in precise English.
Even though the manuscript largely lacks the mechanistic insights how N-glycans regulate and what proteins is most responsible for these cellular phenotypes, the phenomena itself might be informative to the reader of glycobiology and tumor biology.
I think the publication of the manuscript can be considered if the following concerns are addressed.

Major point
1. through the manuscript
GnT-1 and GnT-2 should be GnT-I and GnT-II (Roman, not Arabian)

Authors response: This has been changed throughout the manuscript.

2. The schematic drawing to show N-glycans structures and how GnT-I and GnT-II work on the structures should be briefly presented on Figure 1A for beginner readers.

Authors response: A schematic drawing has been added as Figure 1A. The figure legend was updated, lines 300-303. The figure is stated in the text of the results, lines 292-294.

3. Figure 1D
The samples should be run on the same gel and blotted on the same membrane for each blot.

Figure 1D was changed to Figure 1E due to insertion of the schematic drawing requested by reviewer. The samples were run on the same blot as shown and can also be viewed in the S1_Raw_images file. We have removed lines on blots for clarification.

4. Figure 9A
The image is very poor and the samples should be run on the same gel and blotted on the same membrane for each blot.

The samples were run on the same blot as shown and can also be viewed in the S1_Raw_images file. For clarification purposes, we have removed lines on blots. In some cases, the 2D and 3D samples were separated by standards as shown in figure. The standard was included on blot to illustrate the samples were on the same blot.

5. The authors should more discuss the relevance of their finding to the human clinical situations. For instances, human neuroblastoma is classified into stage 1 to 4 and 4S. Do you have any information about the expression of GnT-I with different stages?

Currently, information is lacking about the expression of GnT- I with the various stages of NB. However, we have elaborated on GnT-I in favorable and unfavorable NB in the 2nd paragraph of the discussion.

"… Again, reinforcing the relative great cell invasiveness of NB_1(-*Mgat1*) cells. Based on our 3D cell invasion studies, NB cells expressing complex N-glycans were less invasive than those expressing exclusively oligomannose. As such, our current results support a past clinical NB study which showed that GnT-V, the enzyme which initiates β1,6-branchpoints, was of higher abundance in favorable stages (1, 2 and 4s) than unfavorable stages (3 and 4) of NB [26]. Thus, it would be of great interest to evaluate whether unfavorable NB patients have lowered expression of GnT-I in tumor tissues compared to those with favorable NB. Further the cell culturing technique appears to have a major impact on interpreting cell invasiveness of NB cells."

Minor point

Lime 108
paladin>paladin

This has been corrected.

Line 132
two dimensional (2D) versus three dimensional (3D) > 2D and 3D
the abbreviations were already defined and not needed here

This has been corrected.

Figure 1C
“NB_1 (Mgat1)” should be “NB_1 (-Mgat1)”

This has been corrected.

Figure 2A
the scale bar should be included into the image

A scale bar (in µm) has been added.

Figure 2B
What is OG? Outgrowth? “Neurite Length” and “Neurite Width” are better.

Outgrowth has been changed to neurite. Also pixels have been converted to µm.

Figure 2D
Need the scale bar.

A scale bar (in µm) has been added.

Figure 3B
Need the scale bar.

A scale bar (in µm) has been added.

Figure 3C Y-axis
What is the unit?

μm^2

Figure 3D
Need the scale bar.

A scale bar (in µm) has been added.

Figure 5A and 5B
Need the scale bar

A scale bar (in µm) has been added.

Figure 5C, Y-axis
Should be "Cell invasion index (ratio to spheroid size)"

Authors reply: The y-axis represents invasion area/sphere area.

Since there appeared to be some confusion, we changed the figure panels. Figure 5B shows how the invasion was calculated (invasion=invasive area – sphere area). The y-axis of figure 5C was changed to invasion area/sphere area. A reference for the analysis has been added in the methods (Berens EB, Holy JM, Riegel AT, Wellstein A. A Cancer Cell Spheroid Assay to Assess Invasion in a 3D Setting. Journal of visualized experiments : JoVE. 2015;(105). Epub 2015/12/10. doi: 10.3791/53409. PubMed PMID: 26649463; PubMed Central PMCID: PMCPMC4692745) (see line 244). Panel D represent plots of sphere area versus invasion area for each cell line to determine Pearson's correlation coefficients between these two variables. The linear fits show a positive and significant correlation of the two variables, and the rationale for representing cell invasiveness of the different cell lines using the invasion/sphere versus cell line plot. The figure legend and results section has been changed accordingly.

Figure 5D, Y-axis
Need the unit pixels?

This panel has been removed and sphere area versus invasion area plot has been added. The units are in µm.

Figure 6
See the comment on Figure 5.

Scale bar has been added. The y-axis of Panels D,E,F have been changed as described above for figure 5. Panels with bar graphs of sphere area versus cell line have been removed.

Figure 7B and 7D, Y-axis
Should be “Gelatinase activity (Mutant/NB_1)

The label of Y-axis has been changed.

Figure 8A
The cell line names are missing.

The names of cell lines have been added.

Figure 9C and 9D, Y-axis
Should be “Band intensity (Mutant/NB_1)” or “Relative expression (Mutant/NB_1)”.

The y-axis was changed to Band intensity (Mutant/NB_1).
